# Acknowledgement to Reviewers of *Toxins* in 2019

**DOI:** 10.3390/toxins12010059

**Published:** 2020-01-19

**Authors:** Toxins Editorial Office

**Affiliations:** MDPI, St. Alban-Anlage 66, 4052 Basel, Switzerland; toxins@mdpi.com

The editorial team greatly appreciates the reviewers who have dedicated their considerable time and expertise to the journal’s rigorous editorial process over the past 12 months, regardless of whether the papers are finally published or not. In 2019, a total of 733 papers were published in the journal, with a median time to first decision of 17.35 days and a median time from submission to publication of 36 days. The editors would like to express their sincere gratitude to the following reviewers for their generous contribution in 2019:
Abbas HamedLewczuk BogdanAbd El Hakim YasminaLewin MattAbdallah Abdelmohsen MohamedLewis RichardAbera BiresawLi DeyaoAbraham AnnLi DongyingAbrahão Nencioni Ana LeonorLiagre BertrandAbrunhosa LuísLiakopoulos VassiliosAbu-Niaaj LubnaLicea AlexeiAccinelli CesareLimatola CristinaAccoroni StefanoLimay-Rios VictorAdamovsky OndrejLimón M. CarmenAdamski ZbigniewLin Cheng-WenAdányi NóraLin Hai-ShuAgriopoulou SofiaLin Hsu YangAizpurua OstaizkaLindstrom Jon MartinAkbar SaminaLittler DeneAKIO NAKANELiu ChangqiAlbrecht Eric A.Liu JiaAlcaide F. J. E.Liu QianqianAlcorn John FLiu YongqiangAloisi Anna MariaLivaniou EvangeliaAl-Saleem FetwehLiverani LilianaAlshannaq AhmadLlewellyn Lyndon E.Alvito PaulaŁobacz AdrianaAman M. JavadŁobocka MalgorzataAmarpuri GauravLoi MartinaAna Juan-GarcíaLópez Miguel ÁngelAnadón ArturoLopez-Jornet PiaAnas Andrea RoxanneLorenz PeterAndersen BirgitteLou JianlongAndersson Håkan SLouzao Ojeda M. CarmenAndjelkovic MirjanaLu SizhaoAndolfi AnnaLu ZhanpingAndrade FernandoLubawy JanAndrade María J.Lucas GuilhermeAndreev DmitriiLuciani GiuseppinaAndreev KonstantinLuedtke BrandonAndreev Yaroslav A.Lukšić BorisANNIBALLI FabrizioLuo MengAnsari RaisLuo ZonghuaAntipova VeronicaMachado RicardoAntonets KirillMackintosh CarolApetrei ConstantinMacRaild ChristopherAppell MichaelMacrander JasonArakawa OsamuMagarlamov Timur Yu.Araujo PedroMajumdar RajtilakArgiles AngelMalemud CharlesAriño AgustínMaletskyi ZakharArmalyte JulijaMallipeddi SrikrishnanArmstrong GlenMammadova-Bach ElminaAshley JonMan AdrianAssunção RicardoMancheño JoséAung Meiji SoeMancianti FrancescaAustin JohnManda GinaAuvray FrédéricManes JordiAvantaggiato GiuseppinaManganelli MauraAvdonin Pavel V.Mantle PeterAviles Francesc XavierMantovani AlbertoAzarnia Tehran DomenicoMantzoukas SpiridonBacchiocchi SimoneManyes LaraBach HoracioMaragos ChrisBae HyunsuMarchini CristinaBae TaeokMarchwińska KatarzynaBae Woong JinMaresca MarcBailly Jean-DenisMaria Teresa M.R.Baker Mark D.Marianna Sanzani SimonaBalan OanaMarie BenjaminBaldin ClaraMarin Daniela ElizaBalestrieri BarbaraMarin Jose JJBanack SandraMarion EstelleBang Moon SukMarkey KevinBani DanieleMarla SandeepBarbano RichardMarque PhilippeBarbieri Silvia S.Marques GuilherminaBarbirz StefanieMartí JoaquínBarisione ChiaraMartin FranckBarrientos Velazquez Ana L.Martín-Ramos PabloBartolotti MassimoMartins CarlaBasak Ajit K.Martins Dra. Ida S. SanoBasti LeilaMartins Mariana RenovatoBaxter Simon WadeMartinson Ellen O.Beard MatthewMärtlbauer ErwinBębenek EwaMary RidoutBeccari GiovanniMascher-Frutschi FabioBechthold AndreasMastanjević KrešimirBejjanki HariniMastanjević KristinaBel YolandaMasuyer GeoffreyBelloso-Uribe KepaMatak IvicaBelykh OlgaMátis GáborBender KellyMattei CesarBerestetskiy AlexanderMatthews Karl R.Berghoff BorkMatttila TapaniBergholz TeresaMavrikou SofiaBerhow Mark A.Maynard JenniferBernardi Aubry FabrizioMazor OhadBerndt Michael C.Mazur-Marzec HannaBernhoft AkselMbata GeorgeBerrada HoudaMcClain MarkBerry JohnMcCormick Susan P.Berthold-Pluta AnnaMcDougal Owen M.Bertuzzi TerenzioMcGowan SheenaBessaire ThomasMcHenry PeterBhatti DanishMcKay Robert MichaelBideshi DennisMcLaughlin JohnBiernasiuk AnnaMcNutt PatrickBilitewski UrsulaMebs DietrichBilliald PhilippeMeca GiuseppeBilska KatarzynaMegighian AramBlackwell BarbaraMehbub Mohammad FerdousBlanco Juan C.Meierhofer DavidBlasi JuanMellies JayBoarescu Paul MihaiMelville StephenBochniarz MariolaMenaldo Danilo LBocian AleksandraMercader JosepBokori-Brown MonikaMercier-Bonin MurielBölcskei KataMerda DéborahBonamore AlessandraMerkley Eric D.Bonar EmiliaMesnage RobinBongiorno DafneMesquita Francisco S.Boomer Jonathan S.Messa PiergiorgioBordes PatriciaMesterhazy AkosBordin LucianaMeulenberg ElineBosso LucianoMeyer Kevin A.Botana Ana MaríaMézes MiklósBotelho Maria JoaoMichailidis GeorgiosBouaïcha NoureddineMichlmayr HerbertBoundy Michael J.Milano SerenaBovee Toine F.H.Minami MasabumiBovera FulviaMinervini FiorenzaBradley Walter G.Mir Sanchis IgnacioBraga Eliane CandianiMiyashita MasahiroBraicu CorneliaMo FeiBraun VolkmarMoar WilliamBräuning BastianModahl CassandraBreidbach AndreasModica Maria VittoriaBrewer MichaelMohamed Salah A.Broadwater MaggieMohamed TarekBrown AngelaMojsoska BiljanaBrown DarenMolgó JordiBrown Euan RMonastyrnaya MargaritaBrown Ronald B.Moncharmont BrunoBrvar MiranMonika UrbaniakBrychcy EwaMontero OlimpioBryden Wayne L.Montes Morán Miguel A.Bryła MarcinMoon YuseokBrzozowska EwaMoore Robert J.Bschleipfer ThomasMorales LauraBubici GiovanniMoreira CristianaBuchanan F. C.Moricoli DiegoBuchanan RobertMossialos DimitrisBui Duc CuongMou XiaozhenBulla Jr LeeMoura AlexandraBullerjahn GeorgeMowe MaxineBurstyn-cohen TalMukhopadhyay SomshuvraBurtey StéphaneMuñoz-Castañeda Juan R.Bzducha-Wróbel AnnaMurphy Caroline SCaballero PabloMurray ShaunaCaballero PrimitivoMuruganandam GopinathCabral CéliaMurugesan G. RajCaceres IsauraMüthing JohannesCai ShuoweiMutiga Samuel K.Caires KyleMylona EleftheriaCajado-Carvalho DanielaNagai SatoshiCalvano Cosima DamianaNagashima HitoshiCalvete JuanNagel RaimundCameán Ana M.Nakashima SouichiCampbell EwanNallasamy PalanisamyCampos Samuel K.Napierala MartaCaro T.M.Napiórkowska-Krzebietke AgnieszkaCarqueijeiro InêsNarra Hema PrasadCarvalho FilipeNarva KennethCastiglioni BiancaNath ArijitCastro Mariana S.Natskoulis PantelisCatanzaro ElenaNatsume MasahiroCatucci LuciaNaumann MarkusCavallari MarcoNaviaux Robert K.Ceci EdmondoNędzarek ArkadiuszCerasino LeonardoNEERUKONDA SABARINATHCerini ClaireNevalainen TimoČerný JiříNg Tzi BunChai Ok HeeNiculita-Hirzel HélèneChakraborty RajaNiedziela TomaszChalivendra Subbaiah C.Nielsen Vance G.Chalyan TatevikNieto FranciscoChamrád IvoNishiyama AkihitoChanda AnindyaNisnevitch MarinaChandrasekaran MurugesanNiu DongyanChang Kuan Y.Noguchi TamaoChang Long-SenNOVAK BarbaraChen ChenNunn PeterChen HaoqingObremski KazimierzChen Jack J.Oda MasatakaChen JianwuOgashawara IgorChen YangOhe KenjiChen ZhaoOkano KunihiroChen ZhaoOkumura ShiroCheng HailiOláh AttilaCheng Juei-TangOlejnik MalgorzataCheng Juei-TangOlejníková PetraCheng Luisa W.Oliveira Paula A.Cheriyath VenugopalanOmur-Ozbek PinarChiang Hsin-IOno HisayaChijiwa TakahitoOshiro NaomasaChippaux Jean-PhilippeOsorio JohanChitalia VipulOstolaza HelenaCho Je-YoelOszust KarolinaCho YukoOyama EtsukoChoi Yoon-EPadula AndrewChoque ElodiePal ChandanChoudhari ShyamalPal DebjaniChoudhary SanjeevPalanisamy SatheeshkumarChoudhury DevasmitaPalma Mario SegioChristina Barja-FidalgoPalmer MichaelChu XianpingPanaccione DanielChudzinski Ana MarisaPanagou E.Z.Chung GeehoonPandey Ramesh PrasadChurro CatarinaPanek JacekCigana CristinaPang ShintaroCinta Pinzaru SimonaPapadimitriou TheodotiCiriaco FulvioPapageorgiou MariaCitores LuciaPark Hee-SooClemetson KJPark Hyun-WooColavita GiampaoloPark JongHoColeman Jeffrey J.Park Kwan-KyuCollett Mark G.Parsons CameronColon Saez JosePatel AmarComte KatiaPatel HardikkumarConverse Paul J.Paterson Gavin K.Cooper RobinPaterson R. Russell M.Corazzari MarcoPatyra EwelinaCortinovis CristinaPauchet YannickCosta João G.Pawlak DariuszCosta Vera MarisaPawlik AnnaCostantino ValeriaPawłowski KarolCosta-Rama EstefaníaPécheur Eve-IsabelleCostello Ben De LacyPeigneur SteveCotella DiegoPejler GunnarCouch James R.Pekár StanislavCozar IPelin MarcoCrickmore NeilPellett SabineCsaba MáthéPepi MilvaCuccioloni MassimilianoPeraica MaiaCukrowska BoženaPereira Ana LuisaCunha SaraPereira Sofia AD’Acquarica IlariaPEREZ-SAYANS GARCIA MARIODabrowski JanuszPerrier JosetteDaehn IlsePerry Michael J.D’Agostino PaulPethő ZoltánDahl Jan-UlrikPetrov Alexey M.Dai YuminPetruk GannaDal Belo Cháriston AndréPettersson HansDall’Acqua StefanoPiastowska-Ciesielska AgnieszkaDall’Asta ChiaraPick FrancesDaly NorellePickett AndyDamron F. HeathPicking William D.Danforth Teresa L.Pietri AmedeoDaniela JakšićPincus SethDatta Gupta AnupamPinheiro JorgeD’auria EnzaPINOTTI LUCIANOD’Auria ValeriaPiotrowska MałgorzataDauwalder OlivierPitterl FlorianDaverey AmitaPlaza-Díaz JulioDavid MichaelPlenis AlinaDavid Michael Z.Podgorski David C.Davletov BazbekPogrmic-Majkic KristinaDe Baere S.Polišenská IvanaDe Bellis LuigiPollard MikeDe Castro Figueiredo Bordon KarlaPoniedzialek BarbaraDe Castro Ramalho TeodoricoPoniedziałek BarbaraDe Cerain Adela LopezPoór MiklósDe Marcos Ruiz SusanaPopham Holly J.De Pauw JokePopoff MichelDe Santis BarbaraPopolo AdaDe Santis PaolaPorcelli FrancescoDebegnach FrancescaPortaro FernandaDefarge NicolasPosselt GernotDegola FrancescaPost AdrianDel Giudice RitaPosthaus HorstDelcourt NicolasPourafshar ShirinDéleris PaulPovey AndrewDellafiora LucaPrentis PeterDellamaria DeboraPrescott John F.Delogu GiovannaPreto MarcoDepa ŁukaszPrevost GillesDerman YagmurPriel AviDevriendt BertProft MarkusD’Haese PatrickProft ThomasDi Giallonardo FrancescaProietti SilviaDi Maro AntimoPrzybilski RitaDias ElsaPrzybylska-Gornowicz BarbaraDiaz DuartePucca Manuela BertoDickert Franz L.Puddick JonathanDidier AndreaPuig Carolina G.Diego-García EliaPulido Olga MDillman AdlerPulvirenti AndreaDinenno Frank A.Puntervoll PålDiochot SylviePusztahelyi TündeDivino-Filho JosèQuereda Juan JoseDmitrenok Pavel S.Racz IldikoDmochowski RogerRadcliff Fiona JaneDobrindt UlrichRadenkovic MiroslavDoerrler WilliamRaikou Vaia D.Domínguez-Pérez DanyRamani KomalDonaldson JanetRampersad SephraDonohue TerrenceRaposio EdoardoDorantes-Aranda Juan JoséRappaz BenjaminDorner Martin B.Rasetti-Escargueil ChristineDosoky NouraRateb Mostafa E.Dou LaetitiaRather GulamDucancel FrédéricRaut SangramDucatelle RichardReed Kent M.Dugon MichelReed Kevin F.M.Durán-Riveroll LorenaReis-Costa PedroDurek ThomasReitzel AdamDutartre PatrickRekand T.Dutertre SebastienResiere DaborDutta SanjuctaRestivo DomenicoDzantiev B. B.Restivo Francesco MariaDziurka MichalRety StephaneEbani Valentina VirginiaReuter KlausEble JohannesReuter TimEbrahim WeaamRibeiro JoãoEdwards Adrianne N.Riccardo Zenezini ChiozziEdwards ChristineRichardson Simon C. W.Ehrenberg MansRichter KatrinEhrich MarionRico-Yuste AlbertoEibl Martha M.Ricupero MicheleElghobashi-Meinhardt NadiaRiehle MichaelElias MiguelRighetti LauraEliopoulos Elias E.Ritieni AlbertoElisabetta CapraiRobertson Joseph W. F.El-Mallakh RifRobinson SamuelElse Terry AnnRocha SoniaEl-Seedi HeshamRodrigues PaulaEndo HarukaRodríguez AliciaErnst KatharinaRodríguez Armando AlexeiErny Guillaume L.Roelants KimEspiña BegoñaRohn SaschaEspinosa Padrón ManuelRohrmann George FEsteve-Romero JosepRojo Maria AngelesEsteves Da Silva Joaquim C.G.Rolland J.l.Estévez JorgeRöltgen KatharinaEtxebeste OierRoncarati DavideEveraert KarelRondoni GabrieleFaassen ElisabethRos UrisFabbro LarelleRosier Peter F. W.Falconer IanRothe HansjörgFaleiro Maria LeonorRoué MélanieFalkinham III Joseph O.Rourke WadeFanelli FrancescaRoy DhruvajyotiFang FrancisRoze Ludmila V.Faraone Mennella Maria RosariaRueter JohnFarka ZdeněkRuiu LucaFarrell HazelRuiz De Escudero InigoFasullo MichaelRuiz-Gómez GloriaFathi Abdallah MohamedRuiz-Velasco VictorFeig AndrewRusso GiulioFeng ZhiweiRybczyńska-Tkaczyk KamilaFernandes Ana S.Ryu ChangseonFernández-Cruz María LuisaRyu Ji-wonFerran AudeRzymski PiotrFerrara MassimoS. Gandhi NehaFerré JuanSabatier Jean-MarcFerrer EmiliaSachkova MariaFerreras José M.Sadhasivam SudharsanFiaccadori EnricoSakai KanaeFiani Maria LuisaSalawu EmmanuelFilipeanu CatalinSalk KateriFink-Gremmels JohannaSalud Bea Roberto De LaFitzgerald DavidSalvi PaoloFlannery BrennaSamdal IngunnFlavell David J.Samuel TemesgenFleury YannickSandhu AmanFlorens NansSandle TimFlores-Guerrero Jose L.Sanny CharlesFogle JonathanSantero EduardoFologea DanielSantini AntonelloFormisano LuigiSantos López Jorge ArturoForoud NoraSantos-Juanes JorgeFoster TimSantulli GaetanoFourtounas KonstantinosŠarkan BojanFox GlenŠarkanj BojanFox Jay W.Sarrocco SabrinaFozo ElizabethSatake MasayukiFrank DaraSato EiichiFreitas MarisaSato EmikoFretté XavierSato ShigeruFrezza ClaudioSatoh HisashiFrucht Steven J.Sauka DiegoFujita MasakiSaviola AnthonyFülöp TiborSavoie Jean-MichelFuly AndréSayyari AminFurukawa TomohiroSchaberle Till F.Fusco VincenzinaScharf Ruediger E.G Gamage DilaniSchaumburg FriederGabrić DraganaSchininà Maria EugeniaGagat MaciejSchirmer BastianGagiu ValeriaSchlager SabineGajęcki MaciejSchmidt MartinGalanis AlexisSchroeder ChristinaGalić VlatkoSchröper FlorianGall BrianSchrum AdamGallardo EugéniaSchubert Wolf-DieterGan RenyouSchwarz PatrickGao XiangSchweiger WolfgangGarber Eric A.E.Sciani JulianaGarcía TaniaScotto D’Abusco AnnaGarcía-Cela EstherSebastian AimyGarcía-Ortega LucíaSedova IrinaGarcia-Robles InmaculadaŠegvić Klarić MajaGarner BiancaSeifert RolandGaro ElianeSelby KatjaGastaldello StefanoSelwood AndyGatti LauraSeubert Erica L.Gaul DavidSeveau StephanieGazerani ParisaSharma NaveenGeffroy-Rodier ClaudeShcherbakova Larisa A.Genitsaris SavvasSheikh AlaullahGentile FabrizioShen JIANQIANGGerard NormaShenoy Avinash R.Gerdol MarcoShephard Gordon S.Gerhard RalfShin-Yi MarzanoGerhardt ChristophShishido Tânia K.Gerin DonatoShoemaker CharlesGerssen ArjenShoguchi EiichiGhosh SumitShrestha RamGiannuzzi LedaSidiropoulos ChristosGiansanti FrancescoSiigur JüriGibble CorinneSilaghi Ciprian N.Gibbs H. LisleSilva AnjanaGiladi MosheSilva CarlaGilbert MatthewSilva Christopher J.Gilli FrancescaSilva Maria JoaoGil-Serna JessicaSimm RogerGiorni PaolaSimon StephanieGkelis SpyrosSimpson TJGomis-Cebolla JoaquinSingh AbhishekGonçalves Rui AlexandreSingh PummiGonzález-Cano RafaelSionov EdwardGonzález-Peñas ElenaSivakumar SushamaGóral TomaszSkalickova SylvieGorson JulietteSkoneczna AdriannaGoswami SandeepSlama PetrGoud K YugenderŚliwińska-Wilczewska SylwiaGraczyk-Jarzynka AgnieszkaSmani YounesGraham KerrSmith DavidGrandemange StéphanieSmith G. JasonGrasso GianvitoSmith Geoffrey R.Green BenSmith JenniferGregory-Eaves IreneSnow Nicholas H.Grieco FrancescoSohl ChristalGris DenisSolanki Manoj KumarGromadzka KarolinaSolcan CarmenGross AaronSolfrizzo MicheleGuerra Alfredo J.Somma StefaniaGuerra ManuelaSondergaard Teis EsbenGuerre PhilippeSong Im SookGuimarães Jorge A.Song JeongminGulic TamaraSoriano José MiguelGuo JingshuSoule TanyaGurban Ana-MariaSousa SandraGutierrez SantiagoSoutourina OlgaGutleb ArnoŠpanič ValentinaGuzhova Irina V.Sparks DarrellH. Krieter DetlefSpiliopoulou IrisHa Tai HwanStana AncaHaesaert GeertStanciu OanaHallegraeff Gustaaf M.Stanford KimHallen-Adams Heather E.Starzak KarolinaHaller Steven T.Statsyuk Natalia V.Hammes Mary S.Stegmayr BerndHamorsky Krystal T.Stella SimoneHan Jong WonStenmark PalHanna AmgadStepanova Nadezhda YuilevnaHanna Ramy M.Stępień ŁukaszHans GuyStewart Linda D.Hanss MichelStixova LenkaHara TakatoshiStoev StoychoHarijan Rajesh KStoian DanaHarris TedStorer Nicholas P.Harrison SabineStrelec IvicaHarvey PetaStremke Elizabeth R.Hasan ShakirStrickland JasonHasegawa MakotoStromberg ZacharyHawlitschka AlexanderStuper KingaHayes FinbarrSu Zheng-YuanHayes WilliamSuda ShoichiroHeaney LiamSuddala Krishna ChaitanyaHeever Johan P. Van DenSugimoto KeiichiroHeit BryanSugita-Konishi YoshikoHellman AmySukdeo NicoleHellmann NadjaSulyok MichaelHenrich Curtis J.Sumarah Mark WHernández-León AlejandroSun YvonneHernández-Martínez PatriciaSundheim LeifHerrera MartaSung Wang ChouHerrero HernándezSurup FrankHerrin DavidSüßmuth RoderichHerring SusanSuzuki ToshiyukiHerzig VolkerŠvedas VytasHevey RachelSwan Sarah C.Hewage Himaya Siddhihalu WickramaSwanson JessicaHewlett ErikSynowiec AgnieszkaHianik TiborSzabó AndrásHietaniemi VeliSzékács InnaHilbeck AngelikaSzilágyi AndrásHildebrandt Jan-PeterSztanke MałgorzataHirabayashi JunSzulc JustynaHo MengfeiTabashnik BruceHoagland PorterTadahiro SuzukiHöckner MartinaTan YanglanHogan NatachaTanaka SatoshiHojnik NatašaTanaka ToshiharuHoogenboom BartTančinová DanaHoonakker MariekeTang AihuaHoward Meredith D.a.Tangni Emmanuel K.Hruska ZuzanaTarozzi AndreaHsu Jih-TayTartaglione LucianaHu ChenlinTashima Alexandre KHU Dong-LiangTaub MaryHu Wei-GangTavcar Kalcher GabrijelaHuang I-HsiuTaylor Erika A.Hübner FlorianTaylor MatthewHuertas-Pérez José FernandoTellez-Isaias GuillermoHughes ScottTerradot LaurentHuguet AntoineTerrazzino SalvatoreHung AndrewTerzi ValeriaHungerford JamesTesh VernonHurley JamesTeter KenHutorowicz AndrzejTetreau GuillaumeIglesias Maria Del RosarioTharad SudaratIguchi TaisenThiel TeresaIijima NoriakiThomsen Mette GoulIkarashi YoshiakiThreadgill MichaelIkeda YasumasaTietze AlesiaIkegaya NaokiTirloni EricaIkonomopoulou Maria PTisi RenataInagaki HidetoshiTittlemier Sheryl A.Iñigo Marcos AlcaldeToca-Herrera JoseInoue SeijiTolleson William.Ireton KeithTolomelli AlessandraIshii KenichiTolosa JosefaIslam M. NurulTomczyk ŁukaszIto YoichiroTopalidou EleniIwano HidetomoTorović LjiljaIwasaki TakashiTorrado EgídioJacevic VesnaTorres JaumeJackson Timothy N. W.Torroba TomasJackson Woo Chit ShingToruńska-Sitarz AnnaJacquin LisaTosti ElisabettaJanda KimToyotome TakahitoJaneczko MonikaTrachtman HowardJanmaat AlidaTrif MonicaJanssen Elisabeth M.-L.Trim CarolJarugula SridharTrim Steven A.Jedziniak PiotrTrinetta ValentinaJembrek Maja JazvinšćakTripathi AshootoshJenkins Jill A.Troutman Jerry M.Jeong Tae-YongTsakiris Ioannis N.Jeßberger NadjaTse William K.F.Ji ShaofeiTsuge HideakiJia LeijieTsumuraya TakeshiJia YingTsutsui ShigeyukiJin ZhaoTubaro AureliaJohnson EricTufféry PierreJohnson Shannon L.Tuffs Stephen W.Jorrin BeatrizTurk TomJosue DelgadoTzeng Shiang-JongJu Jyh-CherngTzika EleniJuan García CristinaUjević IvanaJudson RobertUnković NikolaJunqueira-de-Azevedo InácioUtkin Yuri N.Kahlert StefanVago RiccardoKalb Suzanne R.Valdez JoseKalogianni DespinaVale Carlos Alberto Garcia DoKalsi MeghaVale CarmenKameneva PolinaVale PauloKamennaya NinaValério ElisabeteKamitani ShigekiVamanu EmanuelKANEDA TakeharuVan Sanford DavidKaneko JunVannucchi Maria-GiulianaKang Sang SunVarga Elisabeth (Austria)Kang TaejoonVarga Elisabeth (Denmark)Kang WonkuVarga Matthew G.Kapolos JohnVasas GáborKarlovsky PetrVasconcelos Teresa Maria Pinto Coelho AmadoKarp Barbara IllowskyVasylieva NataliaKarpiński MirosławVathipadiekal VinodKarrow NielVattepu RaviKarus AvoVavitsas KonstantinosKasanen RistoVejdovszky KatharinaKaskel FrederickVelíšek JosefKaszowska MartaVenancio ArmandoKatikou PanagiotaVerdes AidaKato KeitaVerheecke CarolKaukoranta TimoVerma ArjunKellenberger StephanVerpeut JessicaKem William RVidal ArnauKennedy DavidVidic JasminaKerns DavidVilcinskas AndreasKeyel Peter A.Villani AlessandraKhezri AbdolrahmanVinci GiulianaKikuta ShingoVon Ameln-Mayerhofer AndreasKim Byoung Sikvon Reumont BjörnKim Chu-YoungVozzi GiovanniKim Ji HyungVyskočil VlastimilKim Seong-GonVyviurska OlgaKim T. DoohunWakino ShuKim Tae KonWałecka-Zacharska EwaKimura TadashiWalker AndrewKirienko NatashaWalker Roy S. K.Kishikawa NaoyaWan Lam YimKizis DimostenisWang Hao-ChingKlähn StephanWang ShihuaKlassen ViktorWard MicaiahKlotz JamesWarriner KeithKobayashi NobumichiWaskiewicz AgnieszkaKobos JustynaWaśkiewicz AgnieszkaKoch MatthiasWatanabe RyuichiKocira AnnaWaters MatthewKodali MaheedharWeaver MarkKoelman J. H. T. M.Webb RobertKoh Cho YeowWee JosephineKoivisto AriWegrzyn GrzegorzKoksharova Olga A.Weiergräber MarcoKolesárová AdrianaWejnerowski ŁukaszKoliesnik IevgenWeller Michael G.Kollewe KatjaWells TracyKölling-Paternoga RalfWestrick JudyKolobov Alexandr AlexandrovichWhittington CarlKoludarov IvanWiegand ClaudiaKomatsu MasaharuWiesenberger GerlindeKomori YumikoWijeyewickrema LakshmiKonstantinov Spiro M.Willis AnusuyaKool JeroenWilson Brenda AnneKopra KariWilson DavidKorch Shaleen B.Wood SusieKormas Konstantinos Ar.Wood ThomasKosicki RobertWöstemeyer JohannesKostić Aleksandar Ž.Woźny MaciejKőszegi TamásWree AndreasKovač TihomirWrzosek MałgorzataKovbasnjuk OlgaWu Xin-PingKowalska KarolinaWu YinoKozachok SolomiiaWuster WolfgangKozlov SergeyXaplanteri PanagiotaKrisztián BaloghXi XinpingKrock BerndXie PingKropp MartinaXing MinliKrstanović VinkoXing YalanKu SeockmoYabe KimikoKubala LukášYamaguchi YoshihiroKubo TaiYamamoto SuguruKudryashov Dmitri S.Yan JiayueKudva Indira T.Yan ShengminKügler JonasYang FeiKuhnert EricYang Yi-YuanKuligowski MaciejYano ShozoKulma MagdalenaYao YuchengKumamoto EiichiYaoita YasunoriKumar PradeepYe MingyuKumar SujeetYelko Rodríguez-CarrascoKunova AndreaYilmaz NevalKUO Cheng-ChinYoshihisa TOMIOKAKushiro MasayoYoshinari TomoyaKuzmenkov AlexeyYotsu-Yamashita MariLalak-Kańczugowska JustynaYu HaiLam Kwok HoYu Yu-HsiangLamothe ShawnYucel UmutLamy EvelynZacchia MiriamLandschoot SofieZambelli VanessaLang DiZelanis AndreLanot AntoineZeleny ReinhardLappa IliadaZhang ChangyiLarabee Jason L.Zhang FanLattanzio Veronica Maria TeresaZhang KaiLau GeeZhang RuiyongLau-Cam Cesar A.Zhang Si-CaiLaur JoanZhao BoxuanLe Garrec RaphaeleZhao RuimingLeão Pedro N.Zhao RuimingLebar MatthewZheng XintingLee ChanZhou Carol L. EcaleLee Hye SukZhou MeiLee Kyun-WooZhu LuchangLee SangheeZichel RanLee SeungjunZielonka ŁukaszLegáth JaroslavŻielonka ŁukaszLegros ChristianZilberberg NoamLehmann ChristianZimba PaulLemichez EmanuelZingali Russolina BenedetaLemli BeátaZłotkowska DagmaraLemmens-Gruber RosaZobel-Thropp Pamela A.Leo Jack ChristopherZoll JanLeon FranciscoZou XueqingLeon-González Antonio J.Zsarnovszky AttilaLepiarczyk EwaZuliani Juliana PavanLermyte FrederikZupo Valerio

